# Scandinavian Nurses’ Use of Social Media during the COVID-19 Pandemic—A Berger and Luckman Inspired Analysis of a Qualitative Interview Study

**DOI:** 10.3390/healthcare10071254

**Published:** 2022-07-05

**Authors:** Stinne Glasdam, Frode F. Jacobsen, Lisbeth Hybholt, Sigrid Stjernswärd

**Affiliations:** 1 Department of Health Sciences, Faculty of Medicine, Lund University, 222 41 Lund, Sweden; sigrid.stjernsward@med.lu.se; 2 Centre for Care Research, Western Norway University of Applied Services, 5020 Bergen, Norway; ffj@hvl.no; 3 Oslo Campus, VID Specialised University, 0370 Oslo, Norway; 4 Mental Health Services East and Psychiatric Research Unit, Psychiatry Region Zealand, 4200 Slagelse, Denmark; lihy@regionsjaelland.dk

**Keywords:** COVID-19, nurses, nursing practices, Scandinavia, social media

## Abstract

There is a knowledge gap about nurses’ use of social media in relation to and during the COVID-19 pandemic, which demands the upholding of a physical distance to other people, including patients and their relatives. The study aims to explore how nurses in the Scandinavian countries used social media for professional purposes in relation to the first 15 months of the COVID-19 pandemic. Qualitative, semi-structured interviews with 30 nurses in three Scandinavian countries (Denmark, Norway, and Sweden) were conducted. Thematic analyses were made, methodically inspired by Braun and Clarke, and theoretically inspired by Berger and Luckmann’s theory about the construction of social reality. The Standards for Reporting Qualitative Research (SRQR) checklist was used. The results showed that social media was a socialisation tool for establishing new routines in clinical practice. Virtual meeting places supported collective understandings of a specific COVID-19 ‘reality’ and ‘knowledge’ amongst nurses, with the pandemic bringing to the fore the issue of e-professionalism among nurses relating to their clinical practice. However, social media and virtual education were not commonly used in patient contacts. Further, nurses attempted a re-socialisation of the public to proper COVID-19 behaviour through social media. Moreover, blurred boundaries between acting as a private individual and a professional nurse were identified, where ethics of the nursing profession extended to nurses’ private lives.

## 1. Introduction

During the COVID-19 pandemic, nurses stand on precarious ground in an unknown situation, with continuous news feeds that affect both society and the medical field. Nurses’ work situation is affected by the pandemic in multiple ways. Studies show that there is an increased workload based on for instance relocation of colleagues to other units, safety issues, novel and constantly updated work routines, and the absence of caring relatives [1,2,3]. In addition, nurses have difficulties navigating the contradictory needs for both closeness to and distance from patients. Similar to many people during the pandemic, nurses experience an unpredictable future and feelings of worry and insecurity [2,3,4,5]. Several studies show that many nurses experience work-related stress and multiple psychological stressors during the pandemic, leading to burnout [4,5,6,7,8,9,10].

In today’s digitised societies, social media such as Facebook^®^, Instagram^®^, Twitter^®^, and LinkedIn^®^ are sources of information, news, entertainment, networking, connectivity, identity construction, and more, for an increasing number of people and organisations, enabling political, private, and professional uses [11,12,13,14,15]. Social media can be defined as internet-based applications with user-generated content, where individuals and groups create user-specific profiles for a site or app that are designed and maintained by a social media service. Social media services facilitate the development of social networks online by connecting profiles with other individuals and/or groups [16]. The Scandinavian population is a keen user of social media. The three countries’ populations belong to the top 15 countries in the world that use the Internet the most [17]. In Denmark, 95% of the population between the ages of 16 and 74 are online at least once a day, with 83% being online several times a day. In the Danish population, 85% use social media [18], out of which 88% have a profile on Facebook^®^, 80% on Messenger^®^, 50% on Instagram^®^, and 22% on WhatsApp^®^. Among teenagers, 99% have a profile on at least one social media platform. Among 71+ years old, 68% have a profile on at least one social media platform [19]. In Sweden, 83% of Internet users visit social media platforms, with Facebook^®^ being the most used service, especially for people over 25 years, followed by Instagram^®^, which together with Snapchat^®^ is a preferred platform among younger people (≤25 years) [20]. In Norway, Facebook^®^ led the ranking of social networks with 69% of users, with the highest proportion of Facebook users being 40–59 years. Fifteen percent of the population used Pinterest^®^, and 6% and 3% respectively used Twitter^®^ and Instagram^®^. Those platforms are mostly used by people under 40 [21]. Since the COVID-19 pandemic demands physical distance to other people, including patients and their relatives, nurses’ use of social media is a central issue to explore.

Health authorities use social media for informational purposes, with substantial information spread from the World Health Organisation (WHO) (2020) [22] and national health boards [23]. Further examples include the [24,25], which supply Facebook^®^ and Twitter^®^ with resources to encourage the practice of so-called correct COVID-19 behaviour and other relevant COVID-19 information. There is also an increasing use of social media, e.g., Facebook^®^ and LinkedIn^®^ in hospitals as institutions, mainly for public relations, health education, and health-promoting/preventive tasks [26,27,28,29,30,31]. Studies also show that social media use amongst nurses, but also students and faculty members, awakens both curiosity and caution, speaking for the need of education and policy to support the use of social media as a professional tool [32,33]. There is an awareness about professional behaviour and privacy features in social media, with students showing more frequent social media uses for educational purposes than faculty members [32]. As non-appropriate uses can have detrimental effects, however, the development of best practice approaches in both education and professional contexts may be called for to support e-professionalism [32,33], not least relating to the separation of personal and professional roles [34].

Several professional nurse organisations use social media to increase and improve their members’ engagement and communication [35]. In nursing education, nursing students use a broad range of social media such as Facebook^®^, YouTube^®^, WhatsApp^®^, Twitter^®^, Instagram^®^, and LinkedIn^®^ in their learning processes by downloading articles, following shared information and ideas, watching videos, participating in online chats, and completing learning tasks and communicating with peers [36,37]. However, a review shows that several educationalists are reluctant to use social media due to the possibility of added distraction to the students, with social media signaling socialization more than supporting educational purposes [38]. Studies show that the use of social media contributed to procrastination of studies, lower academic success, reduced academic writing skills, and encouraged plagiarism [38]. This indicates that there may be differing views on and experiences relating to the use of social media in nursing education. In a scoping review including 88 studies, Rukavina and colleagues (2021) found that healthcare professionals, including nurses, generally use social media for professional networking, collaboration, education, and training, and for patient education and health promotion. In another review of 10 studies, McKeon and colleagues (2022) [39] showed that especially Facebook^®^ and WeChat^®^ are used by healthcare professionals to deliver health education and facilitate social support related to physical activity and diets. Oliver and colleagues (2022) [40] show how healthcare professionals use Facebook^®^ to facilitate online support groups for relatives of seriously ill patients. Further, Grewal and colleagues (2022) [41] illuminate the use of Twitter^®^ to share and discuss research. However, the review of Rukavina et al. (2021) shows problems in the use of social media among healthcare professionals, such as loosening accountability, compromising confidentiality, blurred professional boundaries, depiction of unprofessional behaviour, and legal issues with disciplinary consequences.

The COVID-19 pandemic, which demands social distance, can be understood as an invitation to nursing students and nurses to use social media and other virtual platforms in their education and professional work. A recent scoping review about nurses’ uses of social media during the COVID-19 pandemic included 11 studies. It shows that nurses use social media as channels to gain information about COVID-19 in relation to the pandemic [42]. An Australian study shows that nurses experience blurred boundaries regarding Facebook^®^ uses, with hazy boundaries between personal and professional uses. There were also concerns with nurses interpreting the conduct of fellow healthcare professionals as unprofessional and crossing the professional boundary if using Facebook^®^ to promulgate anti-vaccination messages and/or give medical advice online [43]. Nurses use social media to share inconsistencies in COVID-19 advice and, in that way, to sharpen attention to possible issues in the management of COVID-19 [42,44]. Further, this recent review shows that nurses use social media for collegial support by highlighting the need for further education and changes in care delivery and redeployment. Social media functioned as profession-promoting channels for nurses through the sharing of heroic self-representations and the acknowledgment of frontline persons in the pandemic by expressing collegial solidarity. Moreover, nurses use social media to display critical working conditions and to challenge or oppose current healthcare management strategies [42,45,46]. This recent review provides snapshots from various regions in the world, in the respective specific local contexts, that are valuable for policy, practice, and pandemic preparedness in the healthcare sector. However, most of the reviewed studies do not have social media as the main focus of research. In addition, it was difficult to separate nurses from healthcare professionals in general. Furthermore, the recent scoping review finds no studies from the Scandinavian countries focusing on nurses’ use of social media relating to the COVID-19 pandemic [42], motivating the current article’s focus on the three Scandinavian countries, i.e., Denmark, Norway, and Sweden. The study aims to explore how nurses in the Scandinavian countries use social media for professional purposes in relation to the first 15 months of the COVID-19 pandemic.

## 2. Materials & Methods

This study was based on qualitative semi-structured interviews of in total 30 nurses in Denmark, Norway, and Sweden. The thematic analysis was methodically inspired by Braun and Clarke (2006) and theoretically inspired by Berger and Luckmann (1966). To enhance the quality and transparency of the study, the Standards for Reporting Qualitative Research (SRQR) checklist was used [47].

### 2.1. Theoretical Framework

Berger and Luckmann (1966) understand ’society’ in terms of an ongoing dialectical process composed of the three moments of externalization, objectivation, and internalization, supporting individuals’ socialization into the society. Socialization refers to the comprehensive and sustained governance that leads individuals into the objective world of (sub)society. Primary socialization occurs through individuals’ growth. Secondary socialization refers to the internalization of institutional or institutional-based “sub-worlds”, such as the healthcare system and related institutions, which are determined by the division of labour and social distribution of knowledge. A key concept in the current study is the socialization of nurses in a suddenly shifting, socially critical situation in the form of the COVID-19 pandemic. An institutional world is experienced as an objective reality, confronting individuals with undeniable facts, external to the individual, and persistent in their reality. Every institution has a motivating and controlling body of knowledge that defines and constructs institutionally appropriate rules of conduct and roles to be played within the context of the institutions in question. Such knowledge represents a body of generally valid truths about reality, where radical deviances from the institutional order come through as a departure from reality. According to Berger and Luckmann (1966), deviances can be pointed out as ignorance, mental disease, or depravity, with consequences for the treatment of the deviant. 

Knowledge is passed on through socialization and internalization. Institutions require legitimations, which permit the individuals to interpret the institutions’ reality. New generations learn these legitimations through socialization, where the institutions’ transmitted knowledge supplies institutionally appropriate rules of conduct, motivating the dynamics of institutionalised conduct and roles to be played [48]. Individuals externalize their own being into the social world and internalize the social world as a reality. The specific societal context, where individuals act and influence their understandings of ‘reality’ and ‘knowledge’ also applies to nurses and their specific context during the pandemic. Individuals embody institutions through constructed roles that correlate with the institutional rules of conduct; roles through which they participate in the social world. Through the internalization of such roles, the same world becomes subjectively real to the individual. Roles, just like institutions, are subject to habitualization and objectification and appear as a body of knowledge with typification of conduct, which are formed through social interaction. 

Institutionalized conduct hence involves thereto congruent roles. Actors typified as role performers will thus be expected to comply with institutionalized roles. Deviance from roles not deemed as optional may be associated with sanctions. Roles thus represent the institutional order. The increasing number and complexity of sub-universes, however, creates a tension between insiders and outsiders, for which the sub-universe is inaccessible if acknowledged as legitimate. Legitimation is achieved through procedures of intimidation (e.g., propaganda, mystification, and manipulation of prestige symbols). While outsiders need to be kept outside and accept the sub-universe as legitimate, insiders must be kept in. Legitimation serves the purpose of constructing and integrating meaning already attached to institutional processes, that is, objectifying meaning. Legitimation thus gives the institutional order a normative dignity, thereby justifying it. Legitimation has both a cognitive and a normative element, implying both ‘knowledge’ and ‘values’, informing the individuals why they should perform some actions over others, and why things are what they are [48]. Compliance and non-compliance with socially defined role standards cease to be optional, though the severity of sanctions may vary from case to case. Berger and Luckmann (1966) show that daily structure makes sense to the individual and gives everyday life meaning because everyday life is apprehended as normal and self-evident. However, if the reality of everyday life becomes problematic and the integration of what the individuals perceive as normal and self-evident no longer holds true, their understanding of everyday life is threatened. Human activity is subject to habitualization and repeated actions become a pattern in individuals [48]. In that light, the pandemic can be regarded as a situation that abolishes everyday life as previously known for nurses, not least in work-related contexts. Moreover, as will be discussed, the social construction of nursing, professionality, and social media influences the actions of the nurses in two ways: 1. In terms of self-censorship, and 2. In terms of self-imposed constraints as to which platform to use and how to use them.

### 2.2. Recruitment

The participants in the current study entailed a purposive sample [49], selected through snowballing and using postings on social media and personal contacts to recruit participants. The inclusion criteria consisted of being a registered nurse who worked during the COVID-19 pandemic. There were no exclusion criteria. Interested nurses contacted the researchers in their specific country. Participants received written and oral information about the study, including information about participation being voluntary, and that they could discontinue their participation without the need to give an explanation or reason. Appointments for the individual interviews were made with the participants. Thirty nurses were included in the study, with 10 nurses from each of the three Scandinavian countries, with nurses working in hospitals, nursing homes, and home-based care services (see background information in Table 1).

### 2.3. Interviewing Process

A semi-structured interview guide was constructed specifically for the current study and translated into the three Scandinavian languages at stake, allowing a relative homogeneous data collection while remaining responsive to the individual participants when exploring their subjective experiences of the topic at hand [50,51]. The interview guide was developed in a consensual process by and amongst all researchers in the team. Consideration was taken to the researchers’ respective knowledge fields focusing on health, media, and communication, and the study’s aim. The guide encompassed four themes: sociodemographic data; general use of social media; private use of social media related to COVID-19; and professional use of social media related to COVID-19. Seven pilot interviews were carried out by each of the seven researchers and discussed. The interview guide was not modified based on these interviews, and the pilot interviews were included in the empirical material. All interviews were conducted via a digital audio-video platform (Zoom). The interviewers performed 3–7 interviews each. The interviews were conducted from April to August 2021, only sound was recorded (external dictaphones), and lasted between 21 and 89 min (average 51 min). The interviewer and the interviewees were unknown to each other prior to the interview. 

### 2.4. Analytic Strategy 

The latent, thematic analysis was methodically inspired by Braun and Clarke (2006) and theoretically inspired by Berger and Luckmann (1966) to illuminate how nurses in the Scandinavian countries used social media during the COVID-19 pandemic. Firstly, all interviews were transcribed verbatim. Secondly, the empirical material was read through in its entirety several times by the researchers for familiarization with its content and to get an overall sense of the whole empirical material. The researchers in the three Scandinavian countries were able to read and understand the empirical material in its totality, as the three different languages are based on a historical language community [52]. Thirdly, the contents of the transcribed, individual interviews were reorganized one interview at a time, using a common matrix. Fourth, the entire empirical material was coded and reorganized in line with the article’s aim, a process for which theoretically inspired questions were used to break down and reduce the amount of the empirical material. The analytical questions were inspired by Berger and Luckmann’s (1966) theory of reality as a social construction, asking what nurses expressed about socialization of the self and others through social media uses related to the pandemic, such as:

- How did the use of social media support the internalization and habitualization of different roles in nurses relating to the pandemic?
- How did the use of social media challenge and/or support the existing social order and reality of nursing practise?
- How did the use of social media support and/or challenge the re-socialization into new behavioural rules related to the pandemic?
- How did the use of social media support and/or challenge roles viewed as non-compliant with the (new) behavioural rules and roles related to the pandemic?

The initial themes were constructed based on the coded material. The themes were reviewed and further developed in a consensual analysis process amongst the authors. Throughout this process, the researchers performed a dialectic process between the constructed themes and the empirical data, ensuring both that the themes reflected the empirical material and that they covered the study’s aim. The researchers initially carried out the described steps separately, then co-jointly. In that way, three themes were defined, refined, and titled [53]: *Social media as a socialization tool for new routines in clinical practice*, *Virtual meeting places supported collective understandings of a COVID-19 ‘reality’* and *‘knowledge’ amongst nurses*, and *Attempts at re-socialization of the public to proper COVID-19 behaviour.*

### 2.5. Ethical Considerations

The study followed the principles of the Helsinki Declaration [54]. It was ethically approved in Norway (no. 343272 and 492729). However, ethical approval was not necessary in Sweden or Denmark as the data collection did not include sensitive personal data. Personal data was handled in accordance with the EU General Data Protection Regulation (GDPR) 2016/679 [55], and Data Protection Acts in Denmark, Norway, and Sweden, respectively [56,57,58]. The project was registered in Personal Data Lund University (PULU), the Norwegian Social Science Data Services (NSD) in Norway, and the Research & Innovation Organisation (RIO) at the University of Southern Denmark in Denmark. Participation was voluntary. Oral and written information about the study was given to all participants, who signed an informed consent prior to the interviews. All data were anonymized and kept inaccessible to anyone other than the researchers. In publications, the study maximizes anonymity with names, person-specific job names, and toponyms being removed [54]. 

## 3. Results

### 3.1. Social Media as a Socialization Tool for New Routines in Clinical Practice 

Nurses used social media as information channels regarding COVID-19 information in professional settings. Social media became a socialization tool helping nurses discuss, take a stance, and internalize the ‘new normal’ of behaviour during the pandemic. It supported nurses’ understandings and performances of their roles as professionals in the medical field during the pandemic. In professional respects, Facebook^®^, for example, facilitated the spread of professional discussions about COVID-19-related behaviour and material on newly defined guidelines in relation to encounters with patients/clients and colleagues.

(Discussion in a Facebook^®^ group) How do you do it in your municipality? Do you have to e.g., hold mother-and-child groups or meet school children? […] it’s good and bad that you share such experiences, […] it has caused some frustration and division in the staff group, […] there were no clear guidelines for health visitors/school nurses and what we were allowed to do.

(DK10)

The professional discussions did not necessarily clarify things for the nurses, as they experienced a lack of coherence between the interpretation of the guidelines and how they were transformed into clinical practice. For example, there was a variety of clinical routines based on the guidelines that depended on the actual setting, which could vary. Further, social media platforms and services such as Messenger^®^ and Facebook^®^ were used as an alert system for colleagues in the face of rapidly changing recommendations, supporting nursing practises in a situation where many habitualized practices were more or less reset and under continuous reconstruction.

I worked in the ambulance service […] We were a large group of people who sought information on how to identify the patients who really needed hospital care or not […] Based on the usual assessment criteria we always use for respiratory problems, we eventually realised that they don’t work. This disease [COVID-19] looks different […] We introduced a safety margin. I found the information on Twitter^®^ from an ear, nose, throat clinic in the UK.

(S3)

The pandemic challenged nurses’ internalized social body of knowledge, consisting of recipes for the mastery of routine problems. Considering the pandemic, nurses used social media to compensate for knowledge gaps and to direct and internalize new routines particular for the medical sub-universe in a situation where COVID-19 was omnipresent. Through this, they influenced basic routines and habits in society, for instance by using COVID-19 protective equipment in encounters with patients or clients. Some nurses, for instance, learned how to remove protective equipment inspired by Facebook^®^ posts including YouTube^®^ films.

In the beginning (of the pandemic), I shared some pictures of someone (friends) making visors when there was a shortage of materials.

(S7)

[Correct way of taking protective equipment off] We looked at different pages on the web—YouTube, among others.

(DK3)

During and before the pandemic, some nurses used YouTube^®^ as a tool to support colleagues in performing technical professional procedures. They used this in combination with dialogues and professional feedback as a way to internalize and incorporate such procedures into habitualized practices.

In this crisis, reality-checks of rules and procedures were explicit and intensive. The individual nurse could improvise reality-maintaining procedures in the face of the crisis. The COVID-19 pandemic was regarded as a collective crisis of reality, where the threat to people’s lives was an overhanging risk. Nurses externalized their use of social media by constructing their own arguments for implementing changes in their practice based on social media information, for instance in relation to the use of protective equipment, such as face masks and when there were disagreements about guidelines. Social media revealed that the new social order in relation to correct COVID-19 behaviour was uncertain, that it was open for interpretation, and thus also for negotiation among the dominant insiders in the medical sub-universe. Facebook^®^, for instance, was used as a channel for critical reflection on protective equipment that nurses were offered and required to use.

(Facebook^®^ group) is called: “I’m a nurse”, I think. There are some who write questions, and then there are one-hundred-and-forty-two comments […] Sometimes, I’ve read the question and wondered if it was something I should just look at more […] Someone had inserted a link where, for example, you are referred to Statens Serum institut or the National Board of Health or WHO […] (We) found some scientific articles (about face masks) […] confronting my manager with (these facts). She took the question further to a corona group in the municipality. Then, we got other kinds of masks. The management also found that you should actually wear both a face mask AND a visor if, for example, you visited someone who had COVID-19.

(DK3)

Nurses used social media to support or establish new routines in clinical practice in the socialization of new generations of nurses and alias nursing students into the medical sub-universe. This supported students’ clinical education during the pandemic. Social media information supported nurses in keeping guidelines for students up-to-date during the new situation with the pandemic, as well as in clarifying what students had to do during their clinical education. Social media information for instance became an inspiration for clinical supervisors during the pandemic, when students were not allowed to have contact with patients. This demanded new ways of internalizing educational clinical practices and of becoming familiar with the logic of the medical sub-universe per se, the different nursing practices, and the relational work associated with this sub-universe. However, nurses experienced inconsistencies between different authorities and institutional recommendations related to nursing students in clinical education as confusing. This pointed to attempts at reconstructing a new social reality in both the medical sub-universe and in society per se.

I’ve been following X University College (on Facebook^®^) because my students come from there. There were lots of doubts in the beginning (of the pandemic). Then you could read at X University College—they wrote one thing and the National Board of Health wrote something else, and then municipality Y came up with its own interpretations […] I was more confused by trying to find out (on Facebook^®^). It was our manager who made the decision that they (students) should be sent home.

(DK3)

Throughout the pandemic, the medical sub-universe’s COVID-19 knowledge and related rules of conduct were not limited to the order of this sub-universe, but spilled over to apply to the social order in society per se. The pandemic created a ‘new normal’ for social behaviour in society in general. It implied that information from social media was relevant for both insiders and outsiders of the medical sub-universe. 

Further, social media, such as Facebook^®^, was used as a new way to inform relatives to patients/residents in nursing homes, as many hospitals and nursing homes were closed for visitors during the pandemic. Social media can thus be understood as channels that were used to support patients’ and relatives’ roles as included in the medical sub-universe despite being excluded from the physical space of for example nursing homes. It can be understood as a socialization into the new pandemic-related rules of behaviour. Further, social media created new collaboration means between relatives and nurses, where the usual ways to connect were disabled due to COVID-19.

Our nursing home has a Facebook^®^ page for relatives to see nice pictures and such things, and that was important during the pandemic.

(N7)

However, social media was also an unused COVID-19 resource among nurses in clinical practice—both before and during the pandemic. At the same time, the use of social media excluded personnel that were non-users from information shared through social media. In that sense, social media was used to socialize personnel into clinical practices, while simultaneously creating a sub-universe within the sub-universe, that is social media insiders and outsiders. This could be interpreted as social media users representing a sub-universe per se within the wider medical sub-universe where nurses (inter)acted, which then also prevailed and/or spilled over into the nurses’ private spheres.

I only use the internet and Snapchat, I’m not on Facebook^®^ or anything like that […] I’ve never used it in a work context.

(N4)

We’ve our own closed Facebook^®^ page in the department. But we’ve actually cut out sharing info there. We started by spreading info there when corona came, but it […] didn’t feel like the right way to do it. We’ve mostly used emails and such things, notices at the department, not Facebook^®^. Not everyone is on Facebook^®^. There were questions about how much you can share. There may be personal things that were not right in retrospect.

(N8)

### 3.2. Virtual Meeting Places Supported Collective Understandings of a COVID-19 ‘Reality’ and ‘Knowledge’ Amongst Nurses 

In this crisis, reality checks of rules and procedures were explicit and intensive, which led to an escalation of nurses’ uses of social media and other virtual platforms. The medico-politically-ruled society set up specific rules and procedures for situations recognized as risking a breakdown of reality. The use of social media had the potential to maintain a collective understanding of what was seen as correct knowledge, at the given time. It also had a social function, supporting nurses in their experiences of work related to COVID-19 and by upholding collegial mingling despite the new conditions with physical distance. Social media facilitated socializing and small talking with colleagues throughout the working days during the pandemic. Such exchanges illustrated the nurses coping with and adapting to a new clinical reality using social media channels. At the same time, opportunities to mingle at a distance were community-creating and consolidated affiliation with both the staff group and the medical sub-universe. Examples included the use of social media to update clinical priorities on the spot and shared related reflections, with nurses sharing knowledge and viewpoints with colleagues. This kind of information exchange primarily happened through chat functions such as Snapchat^®^ and Messenger^®^.

I’d prefer to read things that have been published and scientifically reviewed. But it didn’t seem to exist a year ago, because (COVID-19) had just arrived. Nothing was published. There were anecdotes all the time. And especially a lot from Italy. Many Italian colleagues wrote on Twitter^®^ and various online forums that ‘This is what it looks like with us right now. Prepare for this. These are the lessons we’ve learnt; we’ve discovered that it’s like this’ and so on. It was incredibly valuable.

(S3)

When nurses talked about their social media uses, they often interweaved other kinds of virtual tools such as Zoom^®^ and Teams^®^ in their descriptions. The pandemic could be regarded as an occasion for increased digital competence. It opened up an extensive and sudden need for using such digital tools to keep connected, for instance through virtual personnel meetings, both for informational and educational purposes. These applications illustrated institutions’ adaptation to and adoption of digital tools in the socialization of their personnel during the pandemic. There were challenges associated with virtual meetings if participants did not previously know each other.

(Contact with colleagues via Zoom^®^/Teams^®^) I’ve virtual meetings with the instructors who perform individual staff teaching in the departments (for the rest of the staff) […] I’ve many new colleagues that I’ve hardly seen in real life, only virtually […] I don’t know how they smell, but worse is that micro-mimicry just disappears virtually […] I can’t actually see how many eyes roll when I say something that is controversial.

(DK4)

Education and theoretical training of newly employed personnel was also offered through digital tools. This was done to maintain and consolidate a qualified workforce to handle the various clinical tasks and practices of the medical sub-universe, both during and after the pandemic.

I was sent to a few different places and had to work for a few months. I was, for example, in home nursing and dosed (medication). I was sent to various nursing homes […] I had to take several courses, I had to take financial courses and so on. It was via Skype^®^ that we’d these lectures. […] But there has been a lot of good training, for example in first aid. […] It’s very nice to be able to take this course through Teams^®^.

(N4)

Professional training required maintenance and updating of professional competencies to consolidate the secondary socialisation as a nurse, which under non-pandemic conditions often happened in physical meeting settings. During the pandemic, digital tools were also used for competence development and further training of staff in clinical practice, as a way for nurses to internalize new knowledge.

I’ve (taken part in a lot of information linked to COVID-19 via social media platforms) […] from Region X and the Swedish Public Health Agency. And a vaccine company Z, which has short lectures on Facebook^®^, which we can listen to. We do that a lot. There is usually a lot about TBE (Tick-Borne Encephalitis), but now there has been a lot of corona information. So it has been really good with Facebook^®^!

(S2)

Some nurses appreciated the digital training. Some nurses regarded such digital professional courses as resource-saving in terms of time and money, with good learning outcomes. At the same time, it facilitated access to sufficient work capacity to address clinical challenges, since it took less time for personnel to participate in digital training than, e.g., face-to-face, in more distant locations.

The large professional forum, which the Norwegian Infection Prevention Society holds every autumn, should have been 3 days in X. It became digital, web based. All […] the important professional education has been on the web. It’s really very good. […] It (is good) to have shorter lessons, e.g., one hour per day, instead of three continuous days of education, as you must travel away and spend time travelling.

(N10)

Others found it problematic as interaction was difficult, and technology was experienced as challenging.

We’ve also tried to establish virtual teaching. […] but it doesn’t work properly […] When you’ve a teacher and there are nurses that are individually connected to a computer without a webcam and microphone, or five nurses in a group sitting at a distance with only one webcam […] And if you just suddenly, impulsively want to say: “No, I actually don’t agree with that” or: “May I just ask something?”. Raise your hand virtually and wait in line or write a question in the chat… you can’t do it in the same way (as in real-life encounters).

(DK8)

However, nurses found that digital tools were problematic to use to teach and guide patients. Although patients were part of the nurses’ clinical practice in the medical sub-universe, prerequisites to use social media differ when used for the habitualization of new habits, for instance, or for the integration of new knowledge in patients. This was due to patients being regarded to be in a particularly vulnerable position. This might limit the value of general social media uses for educational and informative purposes depending on patients’ capabilities or nurses’ assessments hereof, and also depending on habitualized preferences.

I don’t think digital courses with (psychiatric) patients are a good idea […] It can be a cross-border experience for patients to participate in group work […] If some of the groups were to be transformed into (WEB-based groups), [patients who are] introverted and have a very hard time sitting in larger forums and opening, it’s completely unrealistic.

(DK4)

### 3.3. Attempts at Re-Socialisation of the Public to Proper COVID-19 Behaviour

Several nurses had internalized and taken it upon themselves to legitimate and support the ‘new normal’ of COVID-19, consisting of the basic rules ‘keep your distance’, ‘wash your hands’, ‘use face masks’, and ‘isolation in case of illness and risk of infection’. Further, several nurses had internalized, legitimized, and supported the medical recommendations about vaccinations. Nurses used Facebook^®^ to externalize and post recommendations and highlight the importance for all to follow the recommendations. Nurses’ uses of social media hence went in line with the dominant institutions’ messages and socialization efforts pertaining to the pandemic and subsequent ‘new behavioural rules’ in the clinical sub-universe and society in general.

I try to share and disseminate quality-assessed information in relation to COVID-19 status, vaccines, infection control and things like that. I don’t want to get into conflict with people I know when it comes to receiving information, but sometimes you must do it […] Especially as a nurse, then you have a duty to provide information, and we really should lead by example. If I see that information is incorrect, then one may have to correct it […] I’m very happy to share the National Institute of Public Health’s pages, because they’re up-to-date, and what is there should be correct.

(N2)

Other nurses actively externalized information by producing material that they shared to the public on social media, such as instructive films, primarily to inform persons viewed as unknowing inside and outside the medical sub-universe. This again supported the dominant institutions’ medico-political discourse on COVID-19 and both healthcare professionals’ and the public’s socialization into promoted behaviour and roles as compliant nurses and solidary citizens, respectively.

The recommendations from the Public Health Agency must be followed. I’ve done that and I think everyone should do it […] to prevent the spread of infection and relieve the healthcare system […] I’ve shared posts where I also wrote […] What is needed is also that healthcare needs to be refurbished, it’s also not possible to say “hold out” all the time because there’ll be no one who will want to work with this (COVID-19).

(S9)

Some nurses were driven by a sense of solidarity, supported by the trade union, with a call to the public to take care of nurses by assuming the obligation to follow the authorities’ recommendations on proper COVID-19 behaviour. They thus contributed to facilitating the public’s socialisation into the authorities’ rules and recommendations.

There was something about ‘the importance of getting vaccinated’. I shared that because I think it gets weird like when people say: “no, I’ll not”. We’ll never end this pandemic if people think it’s everyone’s responsibility (but not theirs).

(S8)

Further, some nurses appeared as private individuals in closed Facebook^®^ groups, actively trying to oppose perceptions of COVID-19 that were not congruent with the dominant medico-political understandings of COVID-19. Nurses perceived members of such groups as suspicious and as holding a distorted perception of reality. Nurses thus acted as outsider rebellions in the closed groups that threatened both the medical sub-universe and the general population. It felt powerful to offer resistance from a rebel insider position in a group as dialogue and discussion were possible. Resistance from an outsider position, on the other hand, was experienced as pointless. At the same time, such penetrating acts of nurses tried to legitimize the medico-political logic of COVID-19 in sub-universes driven by other logics.

Those demonstrations against vaccines and against COVID-19 restrictions, it’s obvious that we’re discussing it. And a little half-laughing at them. Thinking they’re pretty crazy. Because it’s way different from our reality, that you could then imagine that it didn’t exist! So, it ends up with you almost ridiculing them […] It was a way of dealing with it as well.

(S4)

Other nurses acted as rebels by supporting critical voices about the medico-political strategies related to COVID-19, for example by opposing the idea of compulsory vaccination. They hence positioned themselves in an outsider role, which contradicted the logic of the medical sub-universe’s insiders.

I definitely oppose compulsory vaccines, because there can also be side effects. One can always use it as an argument that one should protect others, protect the patients. But if you’re forced to do so, and then you get side effects […] if you haven’t chosen yourself, it becomes an ethical dilemma. […] to find others who had the same opinions as myself online and who supported things that I thought were the right interpretation in the vaccine situation, and that you get ‘likes’, and so. Of course, it also provides motivation.

(N3)

Nurses also remained silent when they encountered critical voices in social media. They assumed that they could not change people’s viewpoints, and they did not want to expose themselves in person. They were loyal insiders in the medical sub-universe. No matter how much they wanted to convert outsiders, they assumed that it was an impossible project that would only lead to unpleasantness in the form of personal attacks on social media and thus an unwanted (public) exposure of themselves.

When people have such definite opinions, you don’t get through no matter what you say. My fingers are itching to answer but then I leave it at that […] and then you get involved in a discussion. You answer and answer, and it becomes more and more unreasonable […] it completely derails in the end, and suddenly there are personal attacks. So better not to get caught up in that.

(N6)

On the contrary, nurses appreciated the opportunity for dialogue, contradicting views, and exchange on social media.

It’s also that when I write something—just this closeness, that I think I can ask a question directly to a person who writes something, and I can also be met by resistance at once. And I think that’s good. If I write something that’s a bit semi-controversial, or that’s not controversial at all, such as someone questioning it, I think that’s fine. Because then I get the opportunity to clarify or to take back what I’ve said, because I may have said something that’s wrong.

(S3)

In general, nurses were aware that both reading and exposing themselves on social media could lead to a mix of being a professional nurse and a private person.

I think one’s private life merges quickly (with work)—perhaps especially here in the corona pandemic, because that one’s work life is an insanely large part of one’s private life, […] when you’re a nurse, it just becomes a big part, where you constantly keep informed, also through (television) NEWS.

(DK1)

Some nurses wanted to avoid exposing themselves or others as it risked merging both the professional sub-universe and the public sphere with the private sphere. Contrarily, others estimated that transparency about the private persona facilitated trust as a professional in social media.

I’ve one and the same account [Twitter^®^ account used for both professional and private purposes]. But I’m myself, so I’ve my name and picture, and a description of who I’m, and my qualifications, so to say. It shows that I’m a nurse, and I can be contacted that way. I use it both to write purely private observations about the world or to comment on political proposals, post pictures of the dog. But even then, to discuss, take part in and discuss, answer, and make posts about current issues concerning health care. […] I think it’s an advantage. It’s a trust capital that follows when you’re not anonymous. Without being a real person so to say.

(S3)

Being a member of a social media platform or exposing oneself on social media were associated with the danger of being recognized and unwillingly sought out by patients or their relatives. Even though nurses were passive or active members on various social media platforms as private individuals, they were aware of the risks of being recognised by others as professionals, i.e., as nurses, which could lead to a merging of their professional and private roles and initiatives relating to COVID-19.

I think a lot about what I share, especially on Facebook^®^. Because I think there are a lot of ‘my’ families who can somehow get to see what I post. So that’s why I’m not one to go in and debate either: Must, shouldn’t be vaccinated. Or: Why should they be tested, all schoolchildren? I don’t go into such debates in the public sector because there I must think that I work as if under a health administration. I must back off, no matter what I think personally.

(DK10)

There was nonetheless a clear dividing line between private and professional roles in terms of respecting professional and ethical rules of conduct.

I’d never share any event from work on social media […] Some do it a lot, but I’m too reserved to do that. […] In part, it’s probably because I don’t want to accidentally say anything that can reveal secrecy, because it’s easier said than done.

(S7)

While some nurses shared COVID-19 information on social media that was in line with both their professional and private opinions, other nurses were careful with sharing information while using one’s nurse title as it had the power to proclaim the medico-political truth and thereby try to educate other people in this understanding of reality. Nurses were aware of the risk of the merging of profession and person through social media. They thought that social media uses could expose such merging, thereby influencing others’ understandings of nurses, not just as a professional title holder but also as a person behind the title.

I’ve been extremely careful about speaking out as a nurse, I’ve only done so a few times. To emphasise facts, and then I’ve also used my professional title. […] to be able to contribute with factual information, and always with references. I’m concerned that what I present as facts should have proper professional references. […] Facebook^®^ is Facebook^®^, you have to take it for what it’s, but of course, someone uses Facebook^®^ almost as a reference work, and it can be dangerous. […] For example, the group “We shouldn’t let our child be infected with COVID-19” […] Initially, a group of people who were opposed to vaccines started it. New “paths” are constantly emerging in that forum, for example with discussions about who should have the vaccine and who shouldn’t. It always takes new paths. I’m not as active there now as I was before, it was especially in the first period after the vaccination began that I was particularly active there.

(N3)

Nurses also reflected upon whether they could stand behind previously shared information or not, as the pandemic and related behavioural rules developed over time.

I shared articles about it, where it was pointed out that mouth protection is a questionable measure, if it helps or not. […] then in retrospect, I realised that “I can’t really stand for this” because it became too political, and I don’t want […] to have a political profile. Rather, I maybe want to stick to a little more medical or fact-based information. In retrospect, I deleted something that I had shared that I felt I couldn’t stand for anymore and the debate has also developed during the past year […] I’ve quite a few international friends who are doctors and researchers and what you thought suddenly became so sensitive depending on what country you came from and how your country had handled it. Sweden was controversial and so they might not really dare to assert their point of view because I also didn’t want to get into a clinch with anyone else.

(S7)

## 4. Discussion

This discussion focuses on two main findings. First, we discuss how the pandemic supports e-professionalism among nurses. Second, we discuss how nurses’ uses of social media were challenged by a fusion of private person and profession. Finally, the study’s methods are discussed.

The findings show that nurses used social media, including both open and closed social network functionalities, and other digital tools to support their clinical practice, for collegial support, supervision, and education, and to extend the medico-political COVID-19 dominant voice within and beyond the medical sub-universe. Social media offers several advantages, such as social support and professional collaboration. In line with this, Rukavina and colleagues (2021) found three key benefits of social media for e-professionalism in their review. These consist of professional networking and collaboration, professional education and training, and patient education and health promotion. However, risks of further reinforcing inequality and the digital divide [20,59] must be prevented through adequate policies, not least when it comes to education online, which the pandemic ‘forced upon’ society where attempts were made to minimize the disruption of students’ learning [59]. Additionally, the current pandemic has been a source of psychosocial stress that has added on nurses’ burden [3,60], where social media can be seen as both a resource facilitating fast access to timely information [61] and as a source of additional stress, e.g., through information overload and risks of misinformation [62,63]. Nonetheless, it is interesting that nurses in the current study did not support the idea of using social media and virtual education with patients in general and during the pandemic. However, some nurses used social media to inform and educate the public, which may encompass potential future patients, as also seen in other studies [64]. This reflects the idea of social media users as prosumers, who can both consume and produce both accurate and misleading health-related information [62,65]. In the current study, the shared and/or produced information largely aligned with dominant discourses on the management of COVID-19. Meanwhile, a study of female scientists’ dissemination of scientific information on YouTube^®^ shows that its impact is limited even though the contents are informative [66]. In the current study, however, nothing can be said about the reception of nurses’ shared information. Statistics also show that different age groups can be more or less keen and frequent users of different social media platforms. Nurses in the current study were between 24 and 61 years old, with a mean age of 42, with both shorter and longer clinical experience. No associations between such variables and the findings can be made in the current study, although it would be of interest to explore in future studies. Rukavina and colleagues’ (2021) review also showed dangers related to social media uses encompassing depictions of unprofessional behaviour, legal issues, and disciplinary consequences. Nurses in the current study, however, showed a high awareness and morale in terms of professional ethics, with the use of social media necessitating respect for professional aspects such as patient confidentiality and the potential power of authority associated with the nursing profession. Therefore, nurses were generally careful about what they shared on social media, especially in the open functionalities of social network platforms, although some nurses came through as more reserved than others in terms of sharing opinions and information through social media. This was partly related to their view on nurses’ professional versus private roles, which is the next discussion point.

The current findings show that there are blurred boundaries between acting as a private individual and a professional nurse. Identification with being a professional nurse functions as an overarching guide for what is acceptable and possible to do as a private individual, also in terms of social media uses [67]. The morale is: Once a nurse, always a nurse, in all situations. It means that the nursing profession’s ethics codex [68,69] goes beyond the profession and the professional framework, which may have consequences for life outside work as a private person. The current findings show that some nurses were active participants in Facebook^®^ groups defined as conspiratorial groups, aiming to raise, breed, and re-socialize the pariahs to good and right COVID-19 understandings and behaviour. In that way, nurses position themselves as nurses on social media with opinions in line with a medico-political understanding and perception of COVID-19 and the ‘new normal’ for proper living [70]. This is put forward as an acceptable way for nurses to behave on social media, from a nurses’ collective perspective [43,71]. Studies show that nurses integrated their professionalism and personality into their practice and their relations to patients [72,73,74]. Nursing is a job, which, in the professionals’ self-understanding, demands that they engage with their unique personality, as well as with human, social, and professional knowledge [75]. The underlying understanding of Western healthcare is based on the idea that good practice depends on personalities [76]. This goes in line with ideas about person-centred care, which focuses on individuals, patients’ narratives, and partnership in care, with nurses using themselves as instruments to achieve this [77,78]. Nonetheless, nurses need to find a balance between being personal without being private in their contacts with patients and their relatives, which the current study also puts in the forefront. The current findings illustrate that nurses’ professionalism is also integrated in their private life and, to a certain extent, frames the possibilities of freedom of speech outside the nursing and professional community [79]. Ashton (2016) [80] supported this by arguing that nurses’ use of social media can cause harm to patients and create legal problems for nurses. The historical image of the subordinate, obedient, and nice nurse seems to remain strong, also in today’s image of and expectations on nurses’ behaviour and appearance [79,81,82,83] both in the medical and public fields. 

This study has both strengths and limitations. Although the sample was relatively limited, the study generated rich data from three Scandinavian countries, which may speak for the findings’ transferability in this geographic area. However, further studies are needed to corroborate and/or expand on the current findings, to strengthen the results’ transferability to other parts of the world, and to explore the influence of sociodemographic factors (e.g., age, work experience, gender) on social media uses in professional contexts. All authors were involved in the analysis process, with a pendulum movement between the transcribed empirical data and the proceeding analysis with the construction of themes until consensus amongst the authors was reached, which strengthened the study’s trustworthiness. The theoretical framework and questions guided the analysis, lifting it to a theoretical level and reducing the potential influence of the researchers’ pre-understandings on the research process. The current findings are nevertheless one amongst other possible interpretations, which was guided by the currently chosen analytical lense. Quotes were used to illustrate the findings, bring out the participants’ voices, and for transparency regarding the authors’ interpretation of empirical data. The analysis also illuminated heterogeneity, tensions, and a richness of experiences and social media uses, which can be further explored in future studies, in more depth. The data collection was performed during the 15–18 months after the pandemic was declared, which contextualizes the findings to that specific period, generating extended uses of digital tools in society, including in healthcare contexts. The results cannot reveal anything about whether or how social media uses and e-professionalism developments are being sustained post-pandemic as new opportunities and tools unfold in the clinical nursing field. Nor can any general conclusions be drawn pertaining to nurses’ use of social media in relation to sociodemographic variables, or specific care contexts, for instance. This calls for further research. 

## 5. Conclusions

Based on thematic analyses of semi-structured interviews with 30 nurses in Denmark, Norway, and Sweden, two main findings have been identified. Firstly, the pandemic brought to the fore the issue of e-professionalism among nurses, in particular, how nurses used social media and virtual tools in their clinical practice, collegial support, and in supervision and education, albeit to a varying extent. A notable exception to e-professionalism is the non-use of social media or virtual education in nurses’ patient contact. At the same time, the non-use of social media may be a sign of professionalism, for instance to avoid confidentiality breaches or use more suitable means to communicate with patients. Furthermore, the non-use of social media can be a consideration taken to issues related to the digital divide, which per se may exclude non-users from whatever happens on and through social media platforms only. Secondly, blurred boundaries appeared in terms of acting as a private person and/or a professional nurse. The saying ‘Once a nurse, always a nurse’ seemed to capture how the nursing profession’s ethics extended to the private life sphere. However, what the consequences of blurred boundaries were varies. Some nurses experienced an obligation to contribute to educating the general public on social media platforms and beyond. Others were hesitant to deal even in private with social media platforms. Several nurses sought information, and some also engaged in conversations, exclusively on platforms that they identified as trustworthy and professional. Despite some individual differences, a more general finding was that nurses were careful in dealing with social media, with professionally based self-applied restrictions limiting their own freedom of speech. Important tensions related to use and non-use of social media have been identified and will be further explored in future studies. Furthermore, policies supporting students’ and nurses’ use of social media in educational and professional contexts are called for to help both students and nurses adopt behaviours that facilitate e-professionalism.

## Figures and Tables

**Table 1:** Description of the participants.

	Denmark	Norway	Sweden	All Countries
**Age** (mean)	24–58 (42)	30–61 (39)	34–53 (45)	24–61 (42)
**Years of experience in nursing** (mean)	1/4-30 + (16+)	8–29 (17)	10–27 (17)	1/4 -30 + (17)
**Highest education level**				
Bachelor	4	5	2	11
Bachelor + specialisation	4	5	8	17
Master	2	0	0	2
**Current work place**				
Hospital	5	3	5	13
Municipality	2	7	3	12
Others	3	0	2	5
**Social media**				
Facebook	10	8	10	28
Instagram	9	6	9	24
LinkedIn	5	0	0	5
Twitter	1	0	1	2
Others	3	10	4	17

## References

[1] Aughterson H., McKinlay A.R., Fancourt D., Burton A. (2021). Psychosocial impact on frontline health and social care professionals in the UK during the COVID-19 pandemic: A qualitative interview study. BMJ Open.

[2] Jørgensen L., Pedersen B., Lerbæk B., Haslund-Thomsen H., Thorup C.B., Albrechtsen M.T., Jacobsen S., Nielsen M.G., Kusk K.H., Laugesen B. (2021). Nursing care during COVID-19 at non-COVID-19 hospital units: A qualitative study. Nord. J. Nurs. Res..

[3] Xu H., Stjernswärd S., Glasdam S. (2021). Psychosocial experiences of frontline nurses working in hospital-based settings during the COVID-19 pandemic—A qualitative systematic review. Int. J. Nurs. Stud. Adv..

[4] Busch I.M., Moretti F., Mazzi M., Wu A.W., Rimondini M. (2021). What We Have Learned from Two Decades of Epidemics and Pandemics: A Systematic Review and Meta-Analysis of the Psychological Burden of Frontline Healthcare Workers. Psychother. Psychosom..

[5] Moore D.J., Dawkins D., Hampton M.D., McNiesh S. (2022). Experiences of critical care nurses during the early months of the COVID-19 pandemic. Nurs. Ethic.

[6] Chen R., Sun C., Chen J.J., Jen H.J., Kang X.L., Kao C.C., Chou K.R. (2021). A Large-Scale Survey on Trauma, Burnout, and Posttraumatic Growth among Nurses during the COVID-19 Pandemic. Int. J. Ment. Health Nurs..

[7] Galanis P., Vraka I., Fragkou D., Bilali A., Kaitelidou D. (2021). Nurses’ burnout and associated risk factors during the COVID-19 pandemic: A systematic review and meta-analysis. J. Adv. Nurs..

[8] Ley C.A., Cintron C.M., McCamant K.L., Karpman M.B., Meisenberg B.R. (2022). COVID-19-related anxieties: Impact on duty to care among nurses. Nurs. Ethic..

[9] Schneider J., Talamonti D., Gibson B., Forshaw M. (2021). Factors mediating the psychological well-being of healthcare workers responding to global pandemics: A systematic review. J. Health Psychol..

[10] Sultana A., Sharma R., Hossain M.M., Bhattacharya S., Purohit N. (2020). Burnout among healthcare providers during COVID-19: Challenges and evidence-based interventions. Indian J. Med. Ethic.

[11] Castells M. (2009). The Rise of the Network Society.

[12] Smith M., Cortez M.F. (2020). Doctors Turn to Social Media to Develop COVID-19 Treatments in Real Time. https://www.bloomberg.com/news/articles/2020-03-24/covid-19-mysteries-yield-to-doctors-new-weapon-crowd-sourcing.

[13] van Dijck J. (2013). The Culture of Connectivity: A Critical History of Social Media.

[14] Ventola C.L. (2014). Social media and health care professionals: Benefits, risks, and best practices. Pharm. Ther..

[15] Vraga E.K., Stefanidis A., Lamprianidis G., Croitoru A., Crooks A.T., Delamater P.L., Pfoser D., Radzikowski J.R., Jacobsen K.H. (2018). Cancer and Social Media: A Comparison of Traffic about Breast Cancer, Prostate Cancer, and Other Reproductive Cancers on Twitter and Instagram. J. Health Commun..

[16] Obar J.A., Wildman S. (2015). Social media definition and the governance challenge: An introduction to the special issue. Telecommun. Policy.

[17] Danish United Nation Association (2020). Andel af Individer Som Bruger Internettet [Proportion of Individuals Who Use the Internet]. https://www.globalis.dk/Statistik/internetbrugere.

[18] Danmarks Statistik (2022). It-Anvendelse i Befolkningen—2021 [IT Use in the Population—2021]. https://www.dst.dk/da/Statistik/nyheder-analyser-publ/Publikationer/VisPub?cid=39431.

[19] Ministry of Culture Denmark (2021). Internetbrug og Sociale Medier 2021 [Internet Uses and Social Media 2021]. https://mediernesudvikling.kum.dk/2021/internetbrug-og-sociale-medier/.

[20] Internetstiftelsen (2018). Svenskarna och Internet [The Swedes and Internet]. https://internetstiftelsen.se/docs/Svenskarna_och_internet_2018.pdf.

[21] Statista (2022). Ranking of Social Networks in Norway as of May 2021, by Market Share. Statista Research Department. https://www.statista.com/statistics/621354/most-popular-social-networks-in-norway-by-page-views/.

[22] World Health Organisation (WHO) (2020). Social Media. https://apps.who.int/dco/strategy/functions/social-media/en/index.html.

[23] Nielsen R.K., Fletcher R., Newman N., Brennen J.S., Howard P.N. (2020). Navigating the ‘Infodemic’: How people in Six Countries Access and Rate News and Information about Coronavirus.

[24] Danish Health Authorities 30th March 2020. Bannere Med Råd og Information om Coronavirus/COVID-19. [Banners with Advice and Information about Coronavirus/COVID-19]. https://www.sst.dk/da/udgivelser/2020/webbannere-med-raad-og-information-om-coronavirus_covid-19.

[25] National Health Institutes (NHI) (2020). COVID-19 Social Media Resources. 24th April. https://www.nih.gov/news-events/covid-19-social-media-resources.

[26] Effah K., Amuah J.E., Dunyo P., Akwada G., Kalmoni Y., Wormenor C.M., Tetteh S., Akakpo P.K. (2021). Raising Funds Through Social Media to Subsidise Cervical Cancer Screening with HPV Testing in Rural Ghana—The Battor Experience. J. Health Care Poor Underserved.

[27] Griffis H.M., Kilaru A.S., Werner R.M., Asch D.A., Hershey J.C., Hill S., Ha Y.P., Sellers A., Mahoney K., Merchant R.M. (2014). Use of Social Media Across US Hospitals: Descriptive Analysis of Adoption and Utilization. J. Med. Internet Res..

[28] Richter J.P., Kazley A.S. (2020). Social media: How hospital facebook activity may influence patient satisfaction. Health Mark. Q..

[29] Ruco A., Baxter N.N., Jacobson J., Tinmouth J., Llovet D. (2022). Using Facebook to promote the uptake of colorectal cancer screening. BMC Public Health.

[30] Sugawara Y., Murakami M., Narimatsu H. (2020). Use of Social Media by Hospitals and Clinics in Japan: Descriptive Study. JMIR Med. Inform..

[31] Van De Belt T.H., Berben S.A., Samsom M., Engelen L.J.L.P.G., Schoonhoven L. (2012). Use of Social Media by Western European Hospitals: Longitudinal Study. J. Med. Internet Res..

[32] Duke V.J., Anstey A., Carter S., Gosse N., Hutchens K.M., Marsh J.A. (2017). Social media in nurse education: Utilization and E-professionalism. Nurse Educ. Today.

[33] Griffin G., Williams N., Bradfield Z., Hauck Y.L. (2021). E-professionalism and social media use amongst nurses and midwives: A cross-sectional study. Nurse Educ. Pract..

[34] Isik B., Jallad S.T. (2019). The potential of social media and nursing education: E-professionalism, nurse educator–learner role, benefits and risks. New Trends Issues Proc. Adv. Pure Appl. Sci..

[35] Wang P., Morgan B., Packard P., Goode V., Tola D. (2020). Maximizing Use of Social Media to Improve Member Engagement in a Professional Organization. AANA J..

[36] Cathala X., Ocho O.N., Watts P.N., Moorley C. (2021). International student nurses’ use of social media for learning: A cross sectional survey. Nurse Educ. Today.

[37] Price A.M., Devis K., LeMoine G., Crouch S., South N., Hossain R. (2018). First year nursing students use of social media within education: Results of a survey. Nurse Educ. Today.

[38] Scott N., Goode D. (2020). The use of social media (some) as a learning tool in healthcare education: An integrative review of the literature. Nurse Educ. Today.

[39] McKeon G., Papadopoulos E., Firth J., Joshi R., Teasdale S., Newby J., Rosenbaum S. (2022). Social media interventions targeting exercise and diet behaviours in people with noncommunicable diseases (NCDs): A systematic review. Internet Interv..

[40] Oliver D.P., Washington K.T., Benson J., White P., Oliver D.C., Smith J.B., Mazur J., Lakew A., Lewis A., Demiris G. (2022). Facebook Online Support Groups for Hospice Family Caregivers of Advanced Cancer Patients: Protocol, Facilitation Skills and Promising Outcomes. J. Soc. Work End Life Palliat. Care.

[41] Grewal U.S., Gupta A., Doggett J., Lou E., Gusani N.J., Maitra A., Beg M.S., Ocean A.J. (2022). Twitter Conversations About Pancreatic Cancer by Health Care Providers and the General Public: Thematic Analysis. JMIR Cancer.

[42] Glasdam S., Sandberg H., Stjernswärd S., Jacobsen F.F., Grønning A.H., Hybholt L. (2022). Nurses’ use of social media during the COVID-19 pandemic—A scoping review. PLoS ONE.

[43] Green J., Petty J., Whiting L., Orr F., Smart L., Brown A.-M., Jones L. (2022). ‘Blurred boundaries’: When nurses and midwives give anti-vaccination advice on Facebook. Nurs. Ethic.

[44] Vindrola-Padros C., Andrews L., Dowrick A., Djellouli N., Fillmore H., Gonzalez E.B., Javadi J., Lewis-Jackson S., Manby L., Mitchinson L. (2020). Perceptions and experiences of healthcare workers during the COVID-19 pandemic in the UK. BMJ Open.

[45] El-Awaisi A., O’Carroll V., Koraysh S., Koummich S., Huber M. (2020). Perceptions of who is in the healthcare team? A content analysis of social media posts during COVID-19 pandemic. J. Interprof. Care.

[46] Forte E.C.N., De Pires D.E.P. (2020). Nursing appeals on social media in times of coronavirus. Rev. Bras. Enferm..

[47] O’Brien B.C., Harris I.B., Beckman T.J., Reed D.A., Cook D.A. (2014). Standards for reporting qualitative research: A synthesis of recommendations. Acad. Med. J. Assoc. Am. Med. Coll..

[48] Berger P.L., Luckmann T. (1966). The social construction of reality. A Treatise in the Sociology of Knowledge.

[49] Palinkas L.A., Horwitz S.M., Green C.A., Wisdom J.P., Duan N., Hoagwood K. (2015). Purposeful Sampling for Qualitative Data Collection and Analysis in Mixed Method Implementation Research. Adm. Policy Ment. Health.

[50] Dejonckheere M., Vaughn L.M. (2019). Semistructured interviewing in primary care research: A balance of relationship and rigour. Fam. Med. Community Health.

[51] McIntosh M.J., Morse J.M. (2015). Situating and Constructing Diversity in Semi-Structured Interviews. Glob. Qual. Nurs. Res..

[52] Lund J. (2006). Det Nordiske Sprogfællesskab [The Nordic Language Community]. Nordisk Försäkringstidskrift, 3. https://nft.nu/sv/det-nordiske-sprogfaellesskab-0.

[53] Braun V., Clarke V. (2006). Using thematic analysis in psychology. Qual. Res. Psychol..

[54] World Medical Association (2013). WMA Declaration of Helsinki: Ethical Principles for Medical Research Involving Human Subjects.

[55] European Union (2016). Regulation (EU) 2016/679 of the European Parliament and of the Council of 27 April 2016 on the Protection of Natural Persons with Regard to the Processing of Personal Data and on the Free Movement of Such Data, and Repealing Directive 95/46/EC (General Data Protection Regulation). http://data.europa.eu/eli/reg/2016/679/oj.

[56] Ministry of Justice and Public Security (2018). Law on the Processing of Personal Data (Personal Data Act) of 15 June 2018.

[57] Ministry of Justice, Denmark (2018). Danish Data Protection Act. Law No 502 af 23/05/2018.

[58] Ministry of Justice, Sweden (2018). Lag (2018:218) Med Kompletterande Bestämmelser Till Eu:s Dataskyddsförordning [Swedish Data Protection Act].

[59] Liu J. (2021). Bridging Digital Divide Amidst Educational Change for Socially Inclusive Learning During the COVID-19 Pandemic. SAGE Open.

[60] Yousaf Z., Nassani A.A., Haffar M. (2021). Destructive Role of COVID-19 Fear on Nurses Performance: Mediating Role of Stress. Nurs. Rep..

[61] Glasdam S., Stjernswärd S. (2022). Ideal types’ strategies related to handling early stages of the COVID-19 pandemic: A thematic analysis of comments from an international survey. Curr. Sociol..

[62] Madathil K.C., Rivera-Rodriguez A.J., Greenstein J.S., Gramopadhye A.K. (2014). Healthcare information on YouTube: A systematic review. Health Inform. J..

[63] Rathore F.A., Farooq F. (2020). Information Overload and Infodemic in the COVID-19 Pandemic. J. Pak. Med. Assoc..

[64] Febrero B., Almela-Baeza J., Ros I., Pérez-Sánchez M.B., Pérez-Manzano A., Cascales P., Martínez-Alarcón L., Ramírez P. (2021). The impact of information and communications technology and broadcasting on YouTube for improving attitude toward organ donation in secondary education with the creation of short films. Patient Educ. Couns..

[65] Eysenbach G. (2008). Medicine 2.0: Social Networking, Collaboration, Participation, Apomediation, and Openness. J. Med Internet Res..

[66] Almela-Baeza J., Febrero B., Pérez-Manzano A., Bonache-Ibáñez A., Ramírez P. (2021). Audiovisual Content to Promote Women Scientists on the YouTube Channels of Spanish Biosanitary Research Institutes. Int. J. Environ. Res. Public Health.

[67] Rukavina T.V., Viskić J., Poplašen L.M., Relić D., Marelić M., Jokic D., Sedak K. (2021). Vukušić Rukavina, T.; Machala Poplašen, L. Dangers and Benefits of Social Media on E-Professionalism of Health Care Professionals: Scoping Review. J. Med. Internet Res..

[68] International Council of Nurses (2012). ICN Code of Ethics for Nurses.

[69] Numminen O., van der Arend A., Leino-Kilpi H. (2009). Nurses’ codes of ethics in practice and education: A review of the literature. Scand. J. Caring Sci..

[70] Glasdam S., Stjernswärd S. (2021). Limit your body area—A COVID-19 mass radicalisation challenging autonomy and basic human rights. Int. J. Hum. Rights Healthc..

[71] Jason R. (2020). Freedom of speech, hate speech, and the nurse practice act. Or. State Board Nurs. Sentin..

[72] Annerstedt C.F., Glasdam S. (2019). Nurses’ attitudes towards support for and communication about sexual health—A qualitative study from the perspectives of oncological nurses. J. Clin. Nurs..

[73] Mason R., Roodenburg J., Williams B. (2020). What personality types dominate among nurses and paramedics: A scoping review?. Australas. Emerg. Care.

[74] Pettersson A., Glasdam S. (2020). Becoming a good nurse—Socialisation of newly employed nurses into the oncological clinic. J. Clin. Nurs..

[75] Callewaert S., Weicher I., Laursen P.F. (2003). Profession og Personlighed—To Sider af Samme Sag? [Profession and Personality—Two Sides of the Same Question?]. Person og Profession—en Udfordring for Socialrådgivere, Sygeplejersker, Lærere og Pædagoger [Person and Profession—A Challenge for Social Workers, Nurses, Teachers and Pedagogues].

[76] Riesman D. (1985). The lonely Mass Person.

[77] Ekman I., Swedberg K., Taft C., Lindseth A., Norberg A., Brink E., Carlsson J., Dahlin-Ivanoff S., Johansson I.L., Kjellgren K. (2011). Person-Centered Care—Ready for Prime Time. Eur. J. Cardiovasc. Nurs..

[78] El-Alti L., Sandman L., Munthe C. (2019). Person Centered Care and Personalized Medicine: Irreconcilable Opposites or Potential Companions?. Health Care Anal..

[79] Hoeve Y.T., Jansen G., Roodbol P. (2014). The nursing profession: Public image, self-concept and professional identity. A discussion paper. J. Adv. Nurs..

[80] Ashton K.S. (2016). Teaching nursing students about terminating professional relationships, boundaries, and social media. Nurse Educ. Today.

[81] Bridges J.M. (1990). Literature review on the images of the nurse and nursing in the media. J. Adv. Nurs..

[82] Erikson C., Stjernswärd S. (2000). Hjältinnan som försvann. En studie av sjuksköterskan i ungdomslitteraturen [The heroine who disappeared. A study of the nurse in youth literature]. Theor. J. Nurs. Theory.

[83] Hallam J. (2000). Nursing the Image: Media, Culture and Professional Identity.

